# Biological and Catalytic Activities of Green Synthesized Silver Nanoparticles from the Leaf Infusion of *Dracocephalum kotschyi* Boiss

**DOI:** 10.1002/gch2.202000018

**Published:** 2020-11-04

**Authors:** Azam Chahardoli, Farshad Qalekhani, Yalda Shokoohinia, Ali Fattahi

**Affiliations:** ^1^ Pharmaceutical Sciences Research Center Health Institute Kermanshah University of Medical Sciences Kermanshah Iran; ^2^ Medical Biology Research Center Health Technology Institute Kermanshah University of Medical Sciences Kermanshah 6715847141 Iran

**Keywords:** antimicrobial effects, blood compatibility, catalytic activity, cytotoxicity, *Dracocephalum kotschyi*

## Abstract

The discovery and development of active compounds to eliminate drug resistance and side effects is a crucial process. In this study, the leaf infusion of *Dracocephalum kotschyi* Boiss as a novel green alternative is used to synthesize silver nanoparticles (Drac‐AgNPs). Antibacterial, cytotoxicity effects, hemocompatibility, and the catalytic properties of these nanoparticles are evaluated. The synthesis of Drac‐AgNPs is confirmed by UV–vis spectroscopy, X‐ray diffraction, Fourier‐transform infrared spectroscopy, and transmission electron microscopy, where Drac‐AgNPs are spherical, with a size range of 5–63 nm. Their IC_50_ values against H1299 and MCF‐7 cell lines are above 50 and 100 μg mL^−1^, respectively. Drac‐AgNPs are effective against an inclusive range of the gram‐positive and gram‐negative bacteria, that is, *Staphylococcus epidermidis*, *Staphylococcus aureus*, *Bacillus subtilis*, *Escherichia coli*, *Serratia marcescens*, and *Pseudomonas aeruginosa*, and a low hemolytic effect makes them an exceptional AgNP with a great hemocompatibility. They show a moderate catalytic‐effect in terms of removing methylene blue, with 67% degradation. Altogether, Drac‐AgNP, as a multi‐tasker material, shows potential for the prevention and treatment of infections and photothermal/chemotherapy of cancers.

## Graphical abstract

In this study, the biological effects of green synthesized silver nanoparticles (19.41 nm) using a leaf infusion of *Dracocephalum kotschyi* (Drac‐AgNPs) are evaluated. Hemocompatible Drac‐AgNPs with a low hemolytic effect are effective against H1299 and MCF‐7cancer cell lines and bacterial strains of *Escherichia coli, Bacillus subtilis*, and *Staphylococcus aureus*. They have a moderate catalytic effect on removing methylene blue.

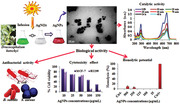

## 1 Introduction

Versatility in plant metabolites and variety in the cornea of green synthesized metallic nanoparticles as a consequence caused colossal attention to the screening of green synthesized nanoparticles. Numerous metallic nanoparticles have been biosynthesized and tested for different applications in the biomedical, cosmetics, and pharmaceutical industries.^[^ ^1^ ^]^ Among them, a massive market of silver nanoparticles (AgNPs) in optical, electrical, chemical, and biological industries caused an urgent for developing a cost‐effective, green, and facile method of synthesis with improved properties of AgNPs at least in one of the tasks that they are applied.^[^ ^2^, ^3^ ^]^

Secondary metabolites are one of the most important metabolites in plants that have received much attention due to their health benefits and effectiveness in preventing various diseases such as cancer and cardiovascular diseases.^[^ ^4^ ^]^ The combination of AgNPs and natural compounds can provide synergistic anticancer effects.^[^ ^5^ ^]^ Furthermore, green synthesized AgNPs, with vigorous antibacterial activity, can be used as an alternative to antibiotics, particularly against multidrug‐resistant bacteria.^[^ ^6^ ^]^ The presence of protein caps on the surface of green synthesized AgNPs leads to their bind and stabilization on the bacterial cell surface and, thus, increases the uptake of the nanoparticles.^[^ ^7^ ^]^

However, for using AgNPs clinically, their blood‐compatibility is the primary unmet need that should be addressed. AgNPs may translocate into the circulatory system through different ways (e.g., systemic ingestion administration and injection), and subsequently, contact and interact with blood cells or plasma proteins, leading to trigger pathophysiologic processes.^[^ ^8^ ^]^ The major cellular components among the circulatory systems are red blood corpuscles (RBCs), which are lysed by exposure to AgNPs.^[^ ^9^ ^]^ The characteristic properties of AgNPs, including size, surface area, coating, surface chemistry, and charge of the NPs, play a significant role in their toxicity and hemolytic activity.^[^ ^7^, ^9^, ^10^, ^11^ ^]^

The catalytic activity for removing pollutants is another property of AgNPs that attracted huge attention. AgNPs have been used for the degradation of organic days, extensively used in textiles, paper, plastic, food, and cosmetic industries.^[^ ^12^, ^13^ ^]^ Methylene blue (MB) is a thiazine dye, used as an anti‐malarial reagent and applied in microbiology, surgery, and diagnostic fields. Overexposure with it leads to nausea, hypertension, hemolysis, and respiratory distress.^[^ ^14^ ^]^

In the present study, we synthesized AgNPs from the leaf infusion of *D. kotschyi* for the evaluation of their multi‐tasker applications. *D. kotschyi* Boiss (known as Badrandjboie of Dennaie and Zarrin‐giah in Iranian folk medicine) belonging to the Labiate family is a wild‐growing Iranian endemic plant.^[^ ^15^ ^]^ This plant used as a traditional folk medicine for disorders of stomach and liver, congestion, and headache^[^ ^16^ ^]^ and also added to tea and yogurt for improving the taste and scent.^[^ ^17^ ^]^ The pharmacological studies on *D. kotschyi* have shown medicinal properties, including analgesic and antispasmodic properties,^[^ ^18^ ^]^ immunomodulatory,^[^ ^19^ ^]^ antihyperlipidemic,^[^ ^20^ ^]^ and cytotoxic effects.^[^ ^18^ ^]^ Compounds such as flavonoids, tannins, saponins, phytosterols, and xanthomicrol (an anticancer compound) have been reported from leaves of *D. kotschyi*.^[^ ^18^, ^21^ ^]^ Taking advantage of this plant, Drac‐AgNPs were synthesized and characterized. Their biological properties (i.e., antibacterial, cytotoxicity, and blood compatibility) were assayed, and their catalytic activity in the removal of MB was investigated.

## 2 Experimental Section

### 2.1 Chemicals

Silver nitrate (AgNO_3_), 3‐(4,5‐dimethylthiazol‐2‐yl)‐2,5‐diphenyltetrazolium bromide (MTT), trypsin, DMSO, Ethylene diamine tetraacetic acid, methylene blue (MB), Triton X‐100, and Mueller Hinton agar and broth were purchased from Sigma Aldrich, USA.

### 2.2 Plant Collection and Extraction

The leaves of the experimental plant (*D. kotschyi*) were collected from the research greenhouse of Razi university campus, Kermanshah, Iran. For removing dust, collected leaves were washed by distilled water, then dried and powdered. 200 mL of 5% powder suspension in double distilled water was boiled for 15 min to prepare an infusion. The infusion was cooled, centrifuged, and filtered by filter paper to get a transparent aqueous extract, then stored at 4 °C for further use.^[^ ^22^ ^]^

### 2.3 Drac‐AgNPs Synthesis and Purification

To synthesis AgNPs, 10 mL of *D. kotschyi* leaf infusion was mixed with 90 mL of an aqueous solution of AgNO_3_ (1mm) and exposed to sunlight in an ambient environment temperature (34.3277 °N and 47.0778 °E). The reaction mixture temperature started at 26 °C and reached 48–50 °C in 45 min under sunlight irradiation. Immediately, the reaction mixture showed a color change from watery yellow to reddish‐brown, confirming the reduction of Ag^+^ to Ag^0^ and the formation of Drac‐AgNPs. The reaction mixture was exposed to sunlight for 2 h; then, it was stored in the laboratory for a further 22 h. After that, the Drac‐AgNPs were collected by centrifuging at 10 000 rpm for 30 min, and the obtained residue was re‐dispersed, washed, and precipitated thrice and dried for further analyses.^[^ ^23^ ^]^ As a control, the experiment was performed at 50 °C without sunlight irradiation to indicate sunlight's role in the formation of nanoparticles.

### 2.4 Physico‐Chemical Characterization of Biogenic Drac‐AgNPs

The reduction of silver ions to NPs was monitored by measuring UV–vis spectra at a resolution of 1 nm in the wavelength range of 200–800 nm and different time intervals (5–120 min) using Shimadzu UV‐2400, Japan. The shape and size of the Drac‐AgNPs were determined using a transmission electron microscopy (TEM) by Zeiss LEO 906 80 kV, Germany. The crystalline structure of the powdered Drac‐AgNPs was determined using an X‐ray diffraction (XRD) (APD 2000, ItalStructures, Italy) in the 2θ range of 20–80° worked at 40 kV and 30 mA with Cu K‐1 radiation. Fourier‐transform infrared spectroscopy (FTIR) spectra of AgNPs and *D. kotschyi* leaf infusion were recorded using an IR prestige‐21 Shimadzu spectrometer using the KBr pellet. The concentration of silver after digestion of the Drac‐AgNPs with HNO_3_ was determined by the atomic absorption spectrometry (AAS) method (AA‐680, Shimadzu, Japan).

### 2.5 Biological Characterization of Drac‐AgNPs

#### 2.5.1 Antibacterial Activity

In the present study, bacteria strains, that is, *Bacillus subtilis* (ATCC 6633), *Staphylococcus epidermidis* (ATCC 12228), *Staphylococcus aureus* (ATCC 43300), *Escherichia coli* (ATCC 25922), *Pseudomonas aeruginosa* (ATCC 27253), and *Serratia marcescens*, (ATCC 13880) were tested for the antibacterial effect of Drac‐AgNPs by well‐diffusion and broth dilution methods. Results were presented as minimum inhibitory concentration (MIC). To this end, the suspension of bacterial strains prepared at final concentrations of 1.5 × 10^8^ CFU mL^−1^ was sub‐cultured uniformly on Muller Hilton agar and broth medium in Petri dishes and 96 well plates, respectively. For the well‐diffusion assay, 100 µL of prepared Drac‐AgNPs was dropped into created wells with a 6.0 mm size on agar plates. For MIC, 50 µL of Drac‐AgNPs was dropped into 96 wells‐plates as serial solutions. After the incubation period at 37 °C and for 24 h, the zone of inhibition and MIC values were determined and compared to controls.

#### 2.5.2 Cytotoxicity Potential of Drac‐AgNPs

The potential Drac‐AgNPs cytotoxicity was studied by MTT assay in MCF‐7 (a human breast cancer) and H1299 (human non‐small cell lung cancer) cell lines. 24 h after seeding 10 000 cells per well in 96 wells plate, wells were treated with 20 µL of either Drac‐AgNPs or plant infusion at different concentrations of 10, 25, 50, 100, and 150 μg mL^−1^. Untreated cells were considered as a control, and experiments were done in three replicates. After treatment for 48 h, 20 µL of MTT (5 mg mL^−1^ in PBS) was added to cells and plates incubated at 37 °C for 3 h. Unreacted MTT was removed, and wells were gently rinsed with 50 mL of PBS. Then 150 mL of DMSO was added in each well to dissolve the formazan crystals. At the end of experiments, the absorbance was recorded with an ELISA plate reader (Bio‐Rad, Model 680, USA) at 570 nm, and the wavelength of 630 nm had been used as a reference. Untreated cells were considered as control. Cell viability was calculated by the following formula:^[^ ^22^ ^]^

(1) $$\begin{array}{c} \text{Percentage}\;\text{of}\;\text{cell}\;\text{viability}\;\left(\%\right)=\frac{\text{Absorbance}\;\text{of}\;\text{sample}}{\text{Absorbance}\;\text{of}\;\text{control}}\times 100 \end{array}$$

#### 2.5.3 Hemocompatibility Assay of Drac‐AgNPs

The effect of Drac‐AgNPs on RBCs was determined using the hemolysis test. Fresh human red blood cells obtained from healthy 24–30 years‐old donors. The blood sample was prepared by centrifugation at 1 500 rpm for 10 min and then was washed thrice by adding normal saline for obtaining a clean pellet. The obtained RBCs were suspended in normal saline (10% v/v). Different concentrations of Drac‐AgNPs (25, 50, 100, 250, and 500 μg mL^−1^) were mixed with RBCs suspension at a ratio of 1:1, and samples were incubated at 37 °C for 60 min, then, all samples were centrifuged at 5 000 rpm for 5 min. Finally, 100 µL of the supernatant of samples was transferred to a 96‐well plate, and their absorbance was measured at 540 nm using the microplate reader. The percentage of RBCs hemolysis was calculated according to the following equation:^[^ ^24^ ^]^

(2) $$\begin{array}{c} \begin{array}{l} \text{Percentage of}\;\text{hemolysis}\;\left(\%\right)=\\ \frac{\left(\text{Sample}\;\text{absorbance}-\text{Negative}\;\;\text{control}\;\text{absorbance}\right)}{\left(\text{Positive}\;\text{control}\;\text{absorbance}-\text{Negative}\;\text{control}\;\text{absorbance}\right)}\times 100 \end{array} \end{array}$$

### 2.6 Catalytic Reduction of Methylene Blue

The MB dye reduction by Drac‐AgNPs was studied by the Nakkala method with minor modifications. For this aim, 10 mL of prepared MB solution (10^−3^ m) was mixed with Drac‐AgNPs (100 μg mL^−1^) and stirred continuously. Then, the reduction of dye was determined by measuring the maximal absorption at regular time intervals using the UV–visible spectrophotometer.^[^ ^23^ ^]^

### 2.7 Statistical Analysis

Statistical analysis was performed using SPSS version16 software (SPSS Inc., Chicago, IL), and the results were evaluated by one‐way ANOVA (Tukey's test). Moreover, results were the average of minimum triplicates. Means with standard errors were calculated, and p < 0.05 was considered statistically significant.

## 3 Results and Discussion

### 3.1 Biosynthesis and Characterization of Drac‐AgNPs

The green synthesis of AgNPs from *D. kotschyi* leaf infusion was confirmed by a color change in infusion solution from yellowish to dark brown after a few minutes of exposure to sunlight. The main metabolic constituents of *D. kotschyi* plant consisting flavonoids (e.g., luteolin‐7‐O‐glucoside, apigenin‐7‐O‐glucoside (Cosmosiin), luteolin 3′‐O‐β‐D‐glucuronide, luteolin, cirsimaritin, iso‐kaempferide, penduletin, xanthomicrol, and calycopterin) and a phenolic compound (i.e., rosmarinic acid),^[^ ^25^ ^]^ can be responsible for the reduction of Ag^+^ ions. Besides, the sunlight radiation as a catalyst is the crucial parameter for the rapid synthesis of stable AgNPs.

#### 3.1.1 UV–Vis Spectra Analysis

**Figure 1:**
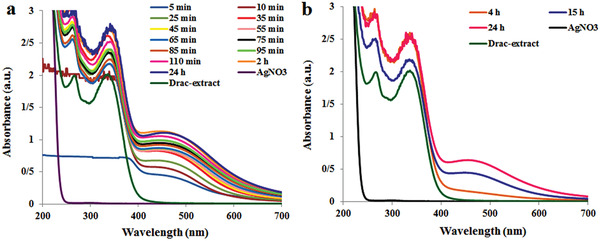
UV‐vis spectra of AgNPs synthesized a) and at room condition b) from *D. kotschyi* leaf extract.

The UV–vis spectra of the AgNO_3_/infusion mixture at different time intervals are shown in **Figure** **1**. The characteristics surface plasmon resonance (SPR) peak for Drac‐AgNPs was monitored by observing the color change and absorbance maxima peak in the range of 420–460 nm as evidence of AgNPs SPR.^[^ ^26^, ^27^ ^]^ The absorption peaks were observed at 447 nm for synthesized Drac‐AgNPs under 2 h of sunlight irradiation. After completion of synthesis, the reaction solution was placed in the laboratory condition for up to 24 h to ensure that the synthesized Drac‐AgNPs would remain stable. After this time, UV–vis analysis was performed again, but no change was observed. (Figure 1a). The broadening of the absorption peaks indicates that the particles are polydisperse. The oscillation of free electrons on the surface of Drac‐AgNPs is a reason for the SPR property of these particles when they align with the irradiated light wavelength in resonance.^[^ ^28^, ^29^ ^]^ Some factors, such as size, shape, and dielectric constant of solutions, determined the position and shape of the SPR peak.^[^ ^30^ ^]^

To evaluate the effect of sunlight, synthesis was performed without sunlight (Figure 1b). It was observed that in the room condition (at a temperature of 50 °C), the color change started from colorless to pale yellow after 4 h and gradually turned to yellowish and dark brown after 15 and 22–24 h, which confirmed the bioreduction of silver ions into AgNPs (Figure 1b). This color change and the formation of nanoparticles were performed slowly in a long time. But under sunlight irradiation, the formation of AgNPs occurred quickly in a few minutes by a color change to dark brown and reached at an optimum and stable level within 2 h. The SPR intensity of Drac‐AgNPs increased with time and stayed constant after 2 h, which demonstrating the complete conversion of Ag^+^ to Ag^0^ (Figure 1a). Therefore, the sunlight mediated synthesis of Drac‐AgNPs is better in terms of the time factor. While at room condition, no SPR was detected for the first 4 h of synthesis. After 15 h, changes in the SPR peak appeared and remained constant after 24 h (Figure 1b).

However, the sunlight mediated synthesis showed a slightly strong SPR compared to synthesis in the room condition due to better electron transfer reaction. The Drac‐AgNPs prepared by sunlight and room condition showed the SPR peak at 447 and 451 nm, respectively (Figure 1). According to AAS analysis, the Ag concentration in 2 300 ppm Drac‐AgNPs was 884.7 ppm, indicating that 38.5% of the synthesized nanoparticles is silver.

#### 3.1.2 XRD Analysis

**Figure 2:**
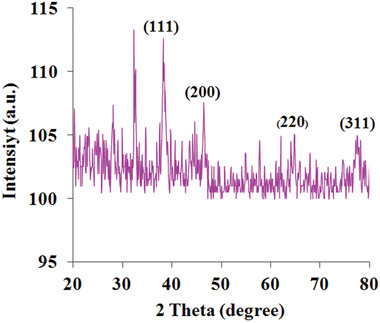
XRD pattern of biogenic Drac‐AgNPs.

The XRD analysis of Drac‐AgNPs is shown in **Figure** **2**. The diffraction intensities of Drac‐AgNPs were recorded in the range of 20–80°. Four intense peaks at 38.30, 46.50, 64.90, and 77.50 respectively indexed to (111), (200), (220), and (311) reflections represent the crystalline structure of Drac‐AgNPs. The average crystallite size of Drac‐AgNPs obtained from the Debye–Scherrer equation (*D* = *kλ*/β cosθ) was 22.5 nm. Besides, three unassigned peaks in the pattern of XRD for Drac‐AgNPs appeared at 28.12, 32.6, and 57.8, which can be relevant to the phytochemical compounds in the Drac‐infusion as a capping agent and stabilizer of the nanoparticle. Some other studies have also reported such unassigned peaks.^[^ ^31^, ^32^ ^]^

#### 3.1.3 FTIR Analysis

**Figure 3:**
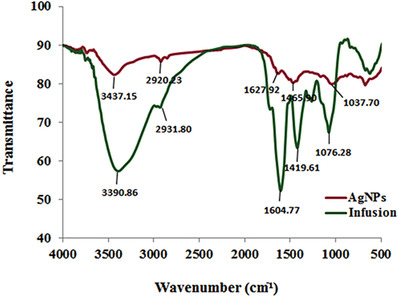
FTIR spectra of *D. kotschyi* leaf infusion and biogenic Drac‐AgNPs.

The role of reducing and stabilizing agents of *D. kotschyi* leave infusion was identified by FTIR analysis (**Figure** **3**). The infrared spectrum showed absorption bands of the infusion at 3 390.86, 2 931.80, 1 604.77, 1 419.61, and 1 076.28 cm^−1^ that shifted to 3 437.15, 2 920.23, 1 627.92, 1 465.90, and 1 037.70 cm^−1^ in Drac‐AgNPs. The absorption bands at 3 437 and 2 920 cm^−1^ corresponded to O—H stretch; H‐bonded of alcohols or phenols, O—H stretch of carboxylic acids, and C—H stretch of alkenes, respectively. It can be clearly seen that the O—H functional group as reducing agents for the formation of Drac‐AgNPs are the primary constitutional components present in the flavonoids and rosmarinic acid from *D. kotschyi* leaf infusion. The peaks at 1 628 cm^−1^ related to N—H bend of amines, and peaks appeared at 1 466 cm^−1^ are attributed to C—C stretch of aromatics or C—H bend of alkanes. The N—H and aliphatic C—H bonds indicate the presence of proteins on the surface of Drac‐AgNPs; proteins can protect these particles from agglomeration and can increase their colloidal stability.^[^ ^33^, ^34^ ^]^ The peak presented at 1 038 cm^−1^ is assigned to C—O stretch of alcohols, carboxylic acids, esters, ethers, and C—N stretch of aliphatic amines. Heterocyclic compounds such as alkaloids and flavones that present in the infusion and acts as capping ligands of the nanoparticles are potential sources of this bound;^[^ ^35^, ^36^ ^]^ they contain a large number of hydroxyl groups that can bind with Ag^+^ to form a complex of Ag^+^/infusion‐compounds. When Ag/infusion‐compounds irradiated in sunlight, the hydroxyl groups present in extract produce hydrated electrons, which reduce the Ag^+^ to Ag^0^.^[^ ^37^, ^38^ ^]^

#### 3.1.4 TEM Analysis

**Figure 4:**
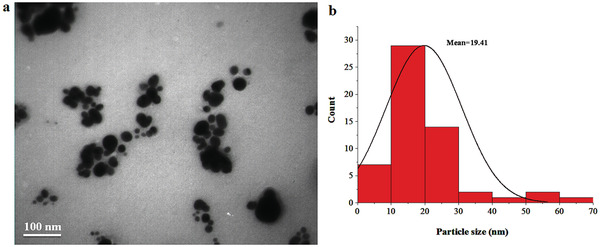
a) TEM image and b) particle size distribution histograms of biogenic Drac‐AgNPs.

The transmission electron microscopy gives information about the size, morphology, and size distribution of Drac‐AgNPs (**Figure** **4**a,b). The TEM image shows that silver nanoparticles are spherical, with a diameter range of 5–63 nm and the average size of 19.41 nm, which is close to the result obtained by XRD.

### 3.2 In Vitro Antibacterial Activity

The antibacterial activity of spherical Drac‐AgNPs was evaluated by well diffusion and broth dilution methods against different bacteria, including *S. epidermidis*, *B. subtilis*, *S. aureus*, *E. coli*, *S. marcescens*, and *P. aeruginosa*. The maximum zone of inhibition in the gram‐negative strain of *E. coli* is 20 mm, with MIC value 3.90 μg mL^−1^. This amount for *S. aureus* and *B. subtilis* (gram‐positive bacteria) is 18 and 15 mm with MIC value 15.63 and 7.81 μg mL^−1^, respectively (**Table** **1**). Drac‐AgNPs also shows full inhibition of growth in *S. epidermidis* strains with MIC of 7.82 μg mL^−1^. Our previous study on antibacterial effects of AgNPs synthesized by *Nigella arevensis* leaf extract with mostly spherical shape and size‐range of 5–100 nm^[^ ^23^ ^]^ showed the inhibition zone of 17 mm and MIC value of 7.82 μg mL^−1^ for *E. coli*, which are significantly weaker than our finding in the current study. In another study, we showed that gold nanoparticles synthesized by *D. Kotschyi* leaf extract also have significant antibacterial effects against *B. subtilis*,^[^ ^22^ ^]^ indicating that *D. Kotschyi* leaf extract has a synergistic impact on antibacterial effects of NPs. *D. Kotschyi* leaf extract mediated NPs have better antibacterial effects comparing to similar NPs from different sources. Among the antibacterial NPs that we have synthesized so far, the Drac‐AgNPs show more antibacterial activity, particularly against *E. coli* and *B. subtilis*, indicating that Drac‐AgNPs can be used in the hospital equipment to prevent common infections and also in food packaging to prevent food poisoning caused by these bacteria. In fact, the prevention of drug‐resistant by silver nanoparticles made them one of the attractive non‐antibacterial reagents and disinfectants for wound care; applying biocompatible AgNPs with wide range of antibacterial effects and can be an alternative way to overcome multi‐drug resistant and hospital infections.^[^ ^6^ ^]^

**Table 1:** Antibacterial activity of Drac‐AgNPs (data of inhibition zone expressed as means ± standard error)

Bacteria strains	Gram positive/negative	MIC [μg mL^−1^]	Zone of inhibition [mm]
*E. coli*	−	3.90	20 ± 0.29
*P. aeruginosa*	−	15.63	13 ± 0.29
*S. marcescens*	−	62.5	13 ± 0.2
*S. aureus*	**+**	15.63	18 ± 0.29
*B. subtilis*	**+**	7.81	15 ± 0.33
*S. epidermidis*	**+**	15.63	13 ± 0.17

### 3.3 Cytotoxic Properties of Drac‐AgNPs

**Figure 5:**
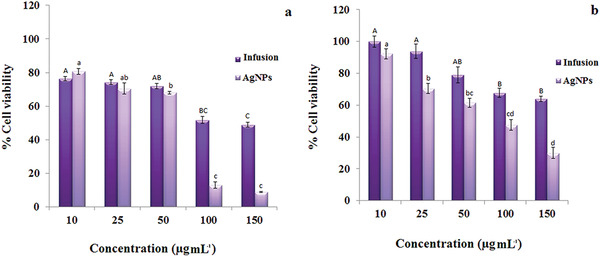
In vitro cytotoxicity potential of biogenic Drac‐AgNPs against a) H1229 cell line and b) MCF‐7cell line at various concentrations. The statistical significance of differences between values was evaluated by one‐way ANOVA (*p* ≤ 0.05).

**Figure 6:**
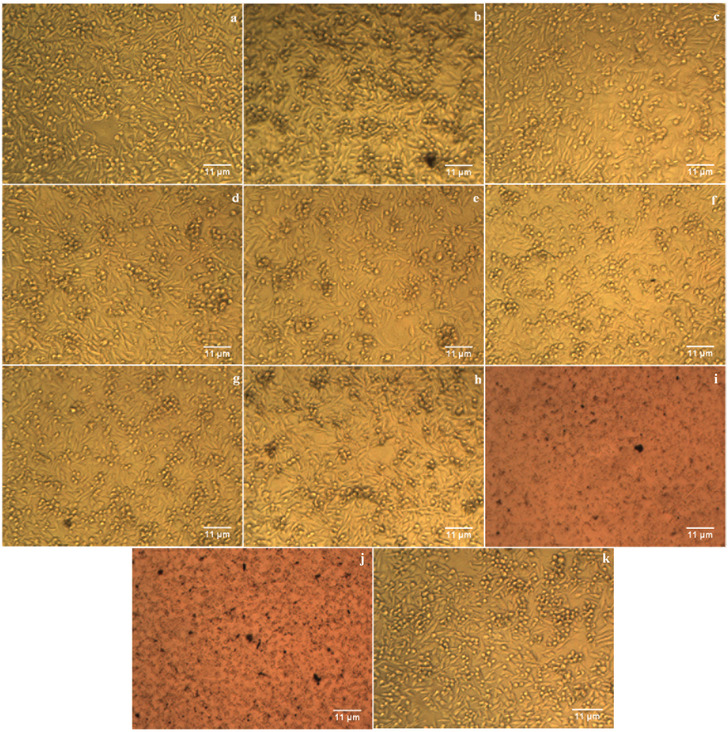
Images of H1299 cell line treated with the leaf infusion of *D. kotschyi* (a–e) and biosynthesized Drac‐AgNPs (f–j), respectively, at concentrations of 10, 25, 50, 100, and 150 μg mL^−1^ and a control (k).

The cytotoxicity of Drac‐AgNPs was evaluated against MCF‐7 and H1299 under MTT assay. Our results recommend that Drac‐AgNPs inhibit the growth of cancer cells significantly in a dose‐dependent manner. The percentage of viability for both cell lines treated with plant extract was significantly higher than that of Drac‐AgNPs (**Figures** **5** **–** **7**). The cell viability of H1299 significantly decreased 48 h post‐treatment with plant extract and Drac‐AgNPs at concentrations of 10, 25, 50, 100, and 150 μg mL^−1^ (Figure 5a,6). The IC_50_ for Drac‐AgNPs was between 50 and 100 μg mL^−1^, while it was 100 μg mL^−1^ for Drac‐infusion. He et al. (2016) indicated a similar inhibitory effect for green synthesized AgNPs on H1299 cells, and cytotoxicity analysis showed a linear dose‐response.^[^ ^39^ ^]^ These cancer cells with specific characteristics show a rapid cell division and a higher rate of metabolism and therefore enhanced the internalization of AgNPs, which likely results in a higher cell death rate. Figure 5b,7 show the cytotoxic effect of Drac‐AgNPs on MCF‐7 cells; a significant difference was observed between Drac‐infusion and Drac‐AgNPs. The IC_50_ of Drac‐infusion and Drac‐AgNPs for MCF‐7 cells were about 150 and 100 μg mL^−1^, respectively.

**Figure 7:**
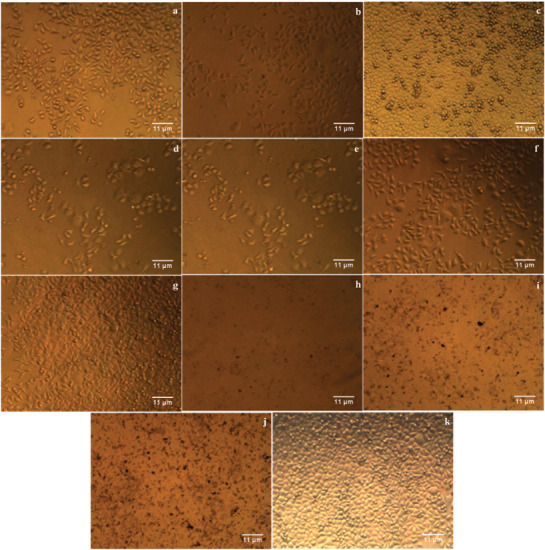
Images of MCF‐7 cell line treated with the leaf infusion of *D. kotschyi* (a–e) and biosynthesized Drac‐AgNPs (f–j), respectively, at concentrations of 10, 25, 50, 100, and 150 μg mL^−1^ and a control (k).

In our previous studies, we obtained significant dose‐response cytotoxic effects of biologically synthesized AgNPs (particle size of 5–100 nm) and AuNPs (paricle size of 3–37 nm) from *N. arevensis* seed and leaf extract,^[^ ^23^, ^40^ ^]^ and AuNPs (particle size of 5–21 nm) from leaf infusion of *D. kotschyi* ^[^ ^22^ ^]^ against MCF‐7 and H1299 cancer cell lines with IC_50_ around 10 μg mL^−1^, which were more cytotoxic than Drac‐AgNPs. Earlier studies have reported that the cytotoxicity effects of green synthesized NPs depend on the size, shape, surface chemistry, and agglomeration state of the nanoparticles and the nature of cells.^[^ ^22^, ^41^, ^42^ ^]^ Based on our previous results about AuNPs from leaf infusion of *D. kotschyi*, the size of synthesized particles was 5–21 nm, which is smaller than synthesized Drac‐AgNPs in the current study with a size range of 5–63 nm. Likely, lower cytotoxicity of Drac‐AgNPs compared to Darc‐AuNPs can be related to their bigger particle size. It is known that the smaller nanoparticles are more cytotoxic due to their easier penetration to cells.^[^ ^41^ ^]^

### 3.4 Hemocompatibility of Drac‐AgNPs

**Figure 8:**
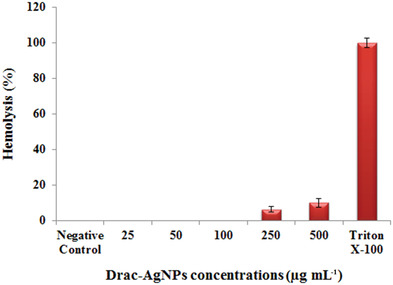
Hemolytic activities of Drac‐AgNPs against human RBCs at various concentrations (25 to 500 μg mL^−1^) and Positive control‐1% TritonX‐100 and Negative control‐ normal saline for 1 h.

Evaluation of the hemolytic potential of biomaterials is one of the critical tests for determining their safety at contact with the bloodstream.^[^ ^43^ ^]^ When hemolysis occurs, the red blood cells are ruptured, and in this condition, the free hemoglobin is released into the blood and may result in adverse health effects.^[^ ^44^ ^]^ Although the hemolytic activity of different nanomaterials has been conducted with hemolysis tests, comparing their results is difficult because of variability in the synthesis, characterization, and hemolysis testing of these materials.^[^ ^43^ ^]^ Based on our results, the biosynthesized Drac‐AgNPs has no toxic effects on RBCs (**Figure** **8**). As shown in Figure 8, 6.4% and 10% of cells were lysed at concentrations of 250 and 500 μg mL^−1^, respectively, in comparison to Triton X‐100 with 100% lysis as a positive control. The significance of our work is more highlighted when we compare our results with the hemolysis effect of AgNPs presented in the literature. Krajewski et al. (2013) and Chen et al. (2015), indicated that chemically synthesized AgNPs with a 10–15 nm size range caused exceeding 60% hemolysis at concentrations of 20 and 30 μg mL^−1^.^[^ ^10^, ^45^ ^]^ Besides, it was shown that citrate capped or free capped AgNPs caused significant hemolysis exceeding 50% at concentrations of 700 μg mL^−1^.^[^ ^43^ ^]^ According to the ASTM E2524‐08 standard, the tested material that causes more than 5% hemolysis leads to damage RBCs. Therefore, biosynthesized Drac‐AgNPs can be a safe and competent candidate for use in medicinal fields, particularly at a concentration fewer than 250 μg mL^−1^.

### 3.5 Catalytic Activity of Drac‐AgNPs

**Figure 9:**
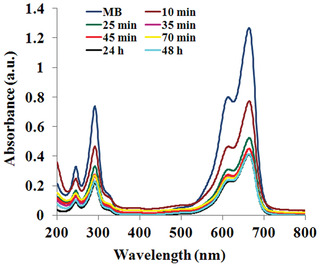
UV–vis spectra of MB at different time intervals after adding biogenic Drac‐AgNPs.

In the present study, the reduction of MB was studied using Drac‐AgNPs as a catalyst at different time intervals in the visible region at room temperature (**Figure** **9**). The absorption peak at 665 nm for MB dye was gradually decreased after the addition of Drac‐AgNPs to the dye. With increasing the exposure time, not only absorbance was slowly reduced, but also λmax was shifted to a higher wavelength. The shift can be relative to a new compound(s), that is, leucomethylene blue resulted from degradation of day.^[^ ^46^ ^]^ The relative absorbance of MB following treatment with Drac‐AgNPs calculated 67% degradation after 48 h (Figure 9). Darc‐AgNPs showed intense catalytic activity comparing to our previous works, where degradation of MB calculated 57% and 44% using biogenic AgNPs and AuNPs synthesized by *N. arvensis* infusion^[^ ^44^, ^47^ ^]^ and 62% for biosynthesized AuNPs using leaf infusion of *D. kotschyi*.^[^ ^22^ ^]^ Therefore, the improved catalytic activity of Drac‐AgNPs can be due to different functional groups or other compounds that are in the leaf infusion of *D. kotschyi* and also decrease in particle size, which will increase the efficiency of the catalyst.^[^ ^48^ ^]^ Furthermore, the phytochemicals presented on the surface of AgNPs may promote the effective adsorption between AgNPs and MB molecules, which can help the reduction processes by attracting them on the catalyst surface by electrostatic interaction.^[^ ^49^ ^]^

## 4 Conclusion

In the present study, we have synthesized stable AgNPs under sunlight irradiation using the leaf extract of *D. kotschyi*. Biosynthesized particles were spherical and small in size. From the FTIR spectra, it is evident that flavonoids, a phenolic compound (rosmarinic acid), and proteins present in Drac‐infusion play a significant role in the stability of Drac‐AgNPs. They show an extreme antibacterial effect with a low cytotoxicity effect. The good catalytic activity of Drac‐AgNPs in the reduction of MB highlights its possible application in removing hazardous environmental pollutants. Furthermore, hemocompatible Drac‐AgNPs with no toxic effects on RBCs showed excellent antibacterial effects. In conclusion, the Drac‐AgNPs with mentioned remarkable properties can be used in biomedical and industrial fields.

## Conflict of Interest

The authors declare no conflict of interest.
